# Coronary anomalies: what the radiologist should know*

**DOI:** 10.1590/0100-3984.2014.0004

**Published:** 2015

**Authors:** Priscilla Ornellas Neves, Joalbo Andrade, Henry Monção

**Affiliations:** 1 Titular Member of Colégio Brasileiro de Radiologia e Diagnóstico por Imagem (CBR), Member of the Society of Cardiovascular Computed Tomography, MD, Radiologist, Hospital Santa Luzia and Hospital do Coração do Brasil (Rede D’Or São Luiz), Brasília, DF, Brazil.; 2 Titular Member of Colégio Brasileiro de Radiologia e Diagnóstico por Imagem (CBR), Member of the Society of Cardiovascular Computed Tomography, MD, Radiologist, Hospital Santa Luzia, Hospital do Coração do Brasil (Rede D’Or São Luiz) and Groups of Radiological Images LifeScan and Padrão Imagens, Brasília, DF, Brazil.; 3 Titular Member of Colégio Brasileiro de Radiologia e Diagnóstico por Imagem (CBR), MD, Radiologist, Hospital Santa Luzia and Hospital do Coração do Brasil (Rede D’Or São Luiz), Brasília, DF, Brazil.

**Keywords:** Coronary anomaly, Coronary computed tomography angiography, Coronary angiography

## Abstract

Coronary anomalies comprise a diverse group of malformations, some of them asymptomatic with a benign course, and the others related to symptoms as chest pain and sudden death. Such anomalies may be classified as follows: 1) anomalies of origination and course; 2) anomalies of intrinsic coronary arterial anatomy; 3) anomalies of coronary termination. The origin and the proximal course of anomalous coronary arteries are the main prognostic factors, and interarterial course or a coronary artery is considered to be malignant due its association with increased risk of sudden death. Coronary computed tomography angiography has become the reference method for such an assessment as it detects not only anomalies in origination of these arteries, but also its course in relation to other mediastinal structures, which plays a relevant role in the definition of the therapeutic management. Finally, it is essential for radiologists to recognize and characterize such anomalies.

## Abstract (pt)

As anomalias coronarianas incluem diversos grupos de malformações, algumas assintomáticas e com curso benigno e outras relacionadas a sintomas, como precordialgia e morte súbita. Podem ser classificadas em: 1) anomalias de origem e de trajeto; 2) anomalias intrínsecas; 3) anomalias de terminação. A origem e o trajeto proximal das artérias coronárias anômalas são os principais fatores prognósticos, sendo o trajeto interarterial considerado maligno, pois está associado a maior risco de morte súbita. A angiotomografia computadorizada tem-se tornado o método de referência para esta avaliação, uma vez que detecta não apenas a anomalia na origem destas artérias, mas também seu trajeto e relação com as demais estruturas mediastinais, que são importantes para a conduta terapêutica. Desta forma, é fundamental que o radiologista saiba reconhecer e caracterizar estas anomalias.

## INTRODUCTION

Coronary anomalies can be found in 0.3% to 5.6% of the population^(1,2)^. In spite of being less frequent as compared with acquired coronary diseases, congenital coronary artery anomalies are associated with morbidity and early mortality in young adults. Reports of sudden deaths occur, in most cases, either during or right after strenuous physical activity^(3)^. It is estimated to be the second most frequent cause of sudden death of cardiovascular origin among athletes, occurring between 12.2% and 17.2% in Europe and in the United States^(4-7)^.

The present review approaches classification, main types, diagnostic methods and treatment of coronary artery anomalies, with emphasis on the condition subgroup with greater clinical repercussion: anomalies of origination and course.

## DISCUSSION

### Normal coronary anatomy

**Figure 1:**
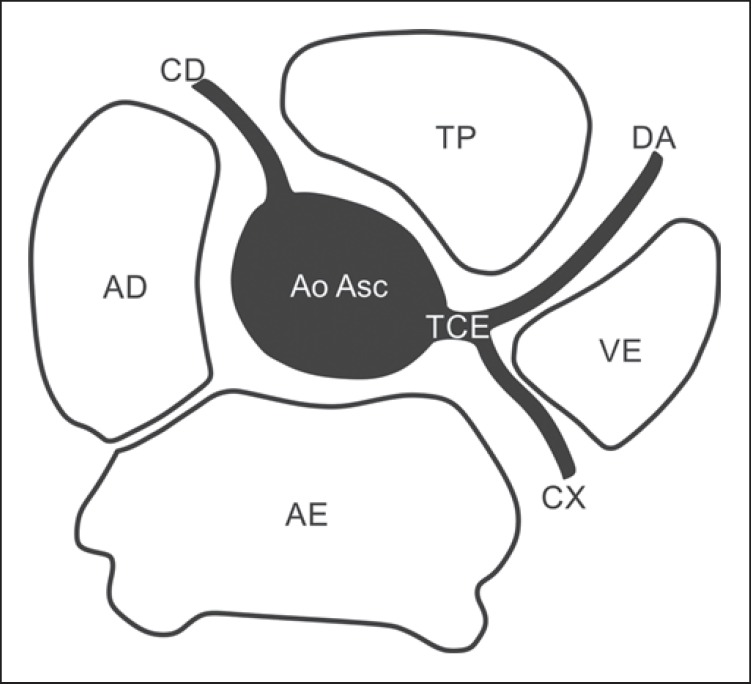
*Normal anatomical origination of coronary arteries*. Right coronary artery (CD) origination from the right coronary sinus travelling in the right atrioventricular groove, and origination of the left coronary trunk (TCE) from the left coronary sinus, bifurcating into descending anterior coronary artery (DA), travelling in the anterior interventricular groove, and circumflex artery (CX) travelling in the left atrioventricular groove. TP, pulmonary arterial trunk; VE, left ventricle; AE, left atrium; AD, right atrium; Ao Asc, ascending aorta.

In the normal coronary anatomy (Figure 1), the right coronary artery originates from the right coronary sinus, and the left coronary artery trunk originates from the left coronary sinus. It posteriorly crosses the pulmonary trunk, bifurcating into anterior descending and circumflex arteries. In approximately 37% of the individuals, there is a trifurcation of the left coronary trunk into anterior descending, circumflex and diagonal coronary arteries or intermediate branch, which irrigates the free lateral wall of the left ventricle^(5)^.

The right coronary artery travels down the right atrioventricular groove. In 50% of individuals, its first branch is the conus branch that supplies the right ventricle outflow tract, and the second branch is the sinoatrial node branch that supplies the sinoatrial node and the right atrium. In 38% of cases, such a branch originates from the left coronary artery, and in 3%, from both arteries. Also, there are branches towards the free wall of the right ventricle, and the branch located in the junction between medial and distal thirds of the right coronary artery is named obtuse marginal artery^(5)^.

In approximately 85% of the individuals, the right coronary artery crosses the *crux cordis* and originates the posterior descending branch (right dominant coronary supply), in 7% to 8% the circumflex artery originates branches to the posterior wall of the right ventricle (left dominant coronary supply), and in 7% to 8% of the cases the posterior interventricular septum is irrigated by branches of the right coronary and circumflex arteries (codominance)^(4,5)^.

The anterior descending artery travels in the interventricular groove and gives off diagonal branches towards the anterolateral wall of the left ventricle.

The circumflex artery travels in the left atrioventricular groove and varies both in size and extent, depending upon the coronary dominance. It gives off one to three marginal branches supplying the free wall of the left ventricle.

### Coronary anomalies classification

The anatomical name of a coronary artery is given according to the dependent territory^(1)^. The right coronary artery is the vessel that supplies the free wall of the right ventricle. The anterior descending artery supplies the anterior wall and the interventricular septum and the circumflex artery supplies the free wall of the left ventricle.

Coronary anomalies may be classified according Angelini et al.^(3)^, as follows: 1) anomalies of origination and course; 2) intrinsic anomalies; 3) termination anomalies (Table 1). Another classification divides coronary anomalies into hemodynamically significant and non-hemodynamically significant. Anomalies classified as hemodynamically significant include: 1) anomalies of origination with interarterial course; 2) anomalous origin in the pulmonary artery; 3) atresias; 4) congenital fistulas^(8)^.

**Table 1:** Coronary anomalies classification (modified from Angelini et al.^(1,3)^).

Anomalies of origination and course
- Coronary ostium in improper coronary sinus: right coronary artery originat-ing from the left coronary sinus, anterior descending and circumflex arter-ies originating from the right coronary sinus, with proximal course anomaly (interarterial, retroaortic, prepulmonic and transseptal )
- Coronary ostium outside the aortic coronary sinus: pulmonary artery, left ventricle, right ventricle, ascending or transverse aorta, etc.
- Single coronary artery
- Absence of the left coronary trunk
- Anomalous location of the coronary ostium in the aortic root: high, low, commissural
Intrinsic anomalies
- Atresia or congenital ostial stenosis, ectasia or aneurysm, hypoplasia or agenesis, etc.
- Intramural course (myocardial bridge) or subendocardial
- Split right coronary artery and anterior descending artery, anomalous origin of the posterior descending artery or first septal branch
Termination anomalies
- Inadequate arteriolar/capillary branching
- Fistulas

**Figure 2:**
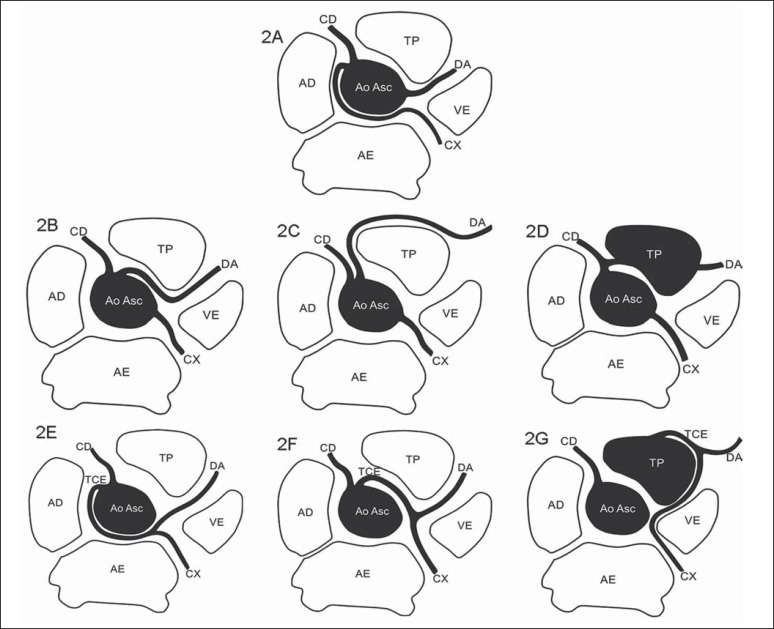
*Anomalous origination of left coronary artery*. **2A:** Anomalous origination of circumflex artery (CX) from the right coronary sinus, with retroaortic course (benign course). **2B:** Anomalous origination of anterior descending artery (DA) from the right coronary sinus with interarterial course (malignant course). **2C:** Anomalous origination of the anterior descending artery (DA) from the right coronary sinus, travelling anteriorly to the pulmonary arterial trunk (TP) (benign course). **2D:** Anomalous origination of anterior descending artery (DA) from the right coronary artery, with transseptal course (benign course). **2E:** Anomalous origination of the left coronary trunk (TCE) from the right coronary sinus, with retroaortic course (benign course). **2F:** Anomalous origination of the left coronary trunk (TCE) from the right coronary sinus, with interarterial course (malignant course). **2G:** Anomalous origination of the left coronary trunk (TCE) from the pulmonary arterial trunk (TP), called ALCAPA (anomalous left coronary artery from the pulmonary artery). CD, right coronary artery; VE, left ventricle; AE, left atrium; AD, right atrium; Ao Asc, ascending aorta.

**Figure 3:**
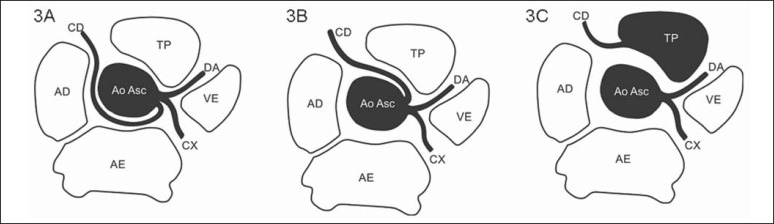
*Anomalous origination of right coronary artery*. **3A:** Anomalous origination of right coronary artery (CD) from the left coronary sinus, with retroaortic course (benign course). **3B:** Anomalous origination of the right coronary artery (CD) from the left coronary sinus with interarterial course (malignant course). **3C:** Anomalous origination of the right coronary artery (CD) from the pulmonary arterial trunk (TP). DA, Anterior descending artery; CX, circumflex artery; VE, left ventricle; AE, left atrium; AD, right atrium; Ao Asc, ascending aorta.

**Figure 4:**
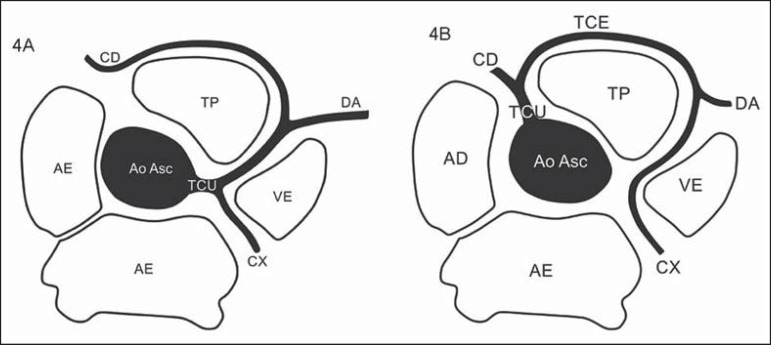
*Single coronary trunk*. **4A:** Single coronary trunk (TCU) originating from the right coronary sinus. **4B:** Single coronary trunk (TCU) originating from the left coronary sinus. CD, right coronary artery; TCE, left coronary trunk; DA, anterior descending artery; CX, circumflex artery; TP, pulmonary arterial trunk; VE, left ventricle; AE, left atrium; AD, right atrium; Ao Asc, ascending aorta.

**Figure 5:**
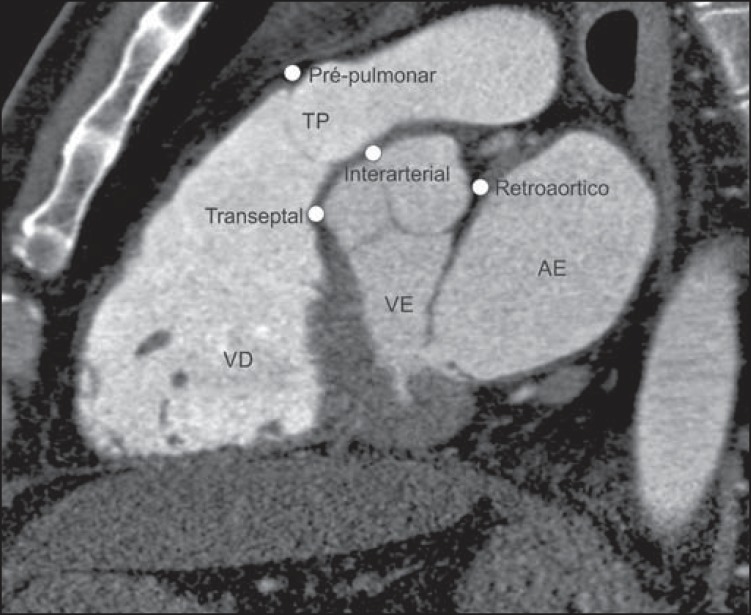
Triple rule-out chest computed tomography angiography with oblique sagittal reconstruction demonstrating four possible proximal courses (pre-pulmonary, interarterial, retroaortic and transseptal) of a coronary artery with anomalous origination. AE, left atrium; VE, left ventricle; VD, right ventricle; TP, pulmonary artery trunk.

The origination and proximal course of the anomalous coronary arteries constitute the main prognostic factors. Figures 2 to 4 demonstrate the main anomalies of coronary arteries origination and course, and Figure 5 depicts a sagittal oblique chest computed tomography image showing the four proximal courses that a coronary with anomalous origination may take, as described below.

#### Interarterial course

A pathway between the aorta and the pulmonary arterial trunk. It is commonly described as “malignant course”, because of the greater risk for sudden death^(8,9)^. It is the most frequent type of hemodynamically significant anomaly^(8)^.

In autopsies performed on athletes who are victims of sudden death caused by coronary anomalies, 80% presented with interarterial course of coronary artery^(6)^.

Several causes are suggested to explain the higher incidence of sudden death in individuals with this type of anomaly. Some authors argue that the vessel with this course would be prone to obstruction during exercise, due to compression by the aorta and the pulmonary artery; but this hypothesis is rejected by some^(8)^. Other commonly associated factors include: 1) acute angle take-off; 2) ostial stenosis/ slit-like ostium; 3) intramural aortic segment^(1,8)^.

#### Retroaortic course

Course between the posterior region of the aorta (noncoronary sinus) and the interatrial septum. No vascular structure is found in this region. In spite of not being associated with the hemodynamic repercussion, it plays a relevant role in cases of cardiac valve surgery. It is generally related to origination anomalies of the left coronary trunk and of the circumflex artery^(8)^. 

#### Prepulmonic course

Anterior course to the right ventricle output tract and to the pulmonary arterial trunk, most commonly associated with anomaly of the left coronary trunk. It may be associated with angina, but generally with no hemodynamic repercussion. It is commonly found in cases of Fallot’s tetralogy^(8)^.

#### Transseptal course

Intramural course in the interventricular septum, considered as being “benign”, and should be differentiated from the interarterial pathway, which is not surrounded by the myocardium, with a more cranial location, above the pulmonary valve; and may present with a stenotic, slit-like ostium^(8)^. The most frequently found anomalous coronary arteries with transseptal pathway are the left coronary trunk and the anterior descending artery^(8)^.

The main types of origination and course anomalies are highlighted, according to the classification proposed by Angelini et al.^(3)^ (Table 1), as follows. 

### Absence of the left coronary trunk

**Figure 6:**
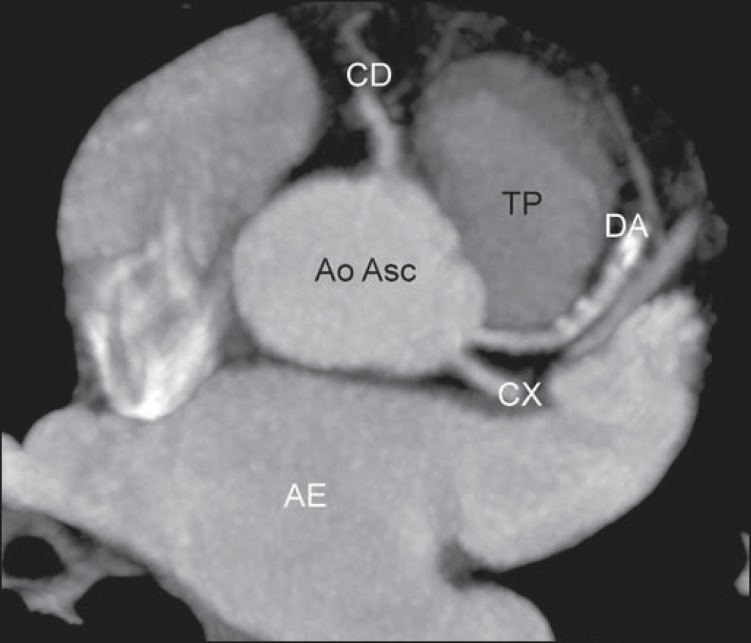
*Absence of left coronary trunk*. Axial coronary computed tomography angiography and MIP reconstruction showing the origination of the anterior descending artery (DA) and circumflex (CX) artery directly from the left coronary sinus. Ao Asc, ascending aorta.; CD, right coronary artery; AE, left atrium; TP, pulmonary artery trunk.

Separated originations of the anterior descending and circumflex arteries are not frequently found (0.4%). They may cause difficulties in catheterization during angiography, but they allow for the development of collateral circulation in the event of proximal obstruction in one of those vessels^(2)^. It is associated with a higher incidence of myocardial bridging and left dominance^(4)^ (Figure 6).

### Anomalous location of the coronary ostium in the aortic root

#### High origin

**Figure 7:**
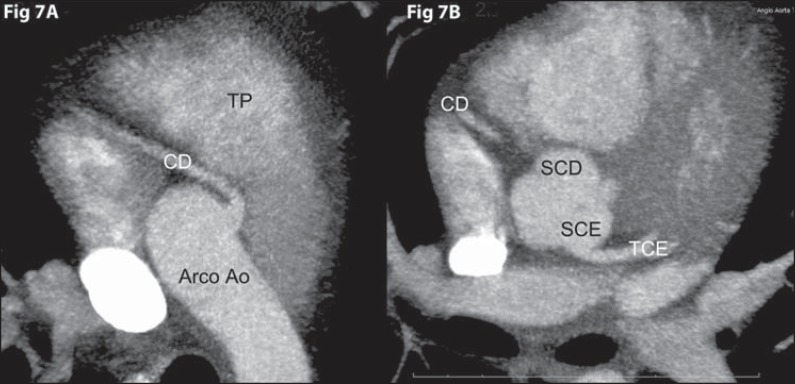
*High origin of right coronary artery*. **Fig 7A:** Axial computed tomography angiography with MIP showing high origin of right coronary artery (CD) from the aortic arch (Arco Ao). **Fig 7B:** Axial coronary computed tomography angiography with MIP showing right coronary sinus (SCD) without coronary artery originating from it. TP, pulmonary artery trunk; SCE, left coronary sinus; TCE, left coronary trunk.

It is defined as origin of a coronary artery or left coronary trunk more than 1 cm above the sinotubular junction^(8)^. It usually does not present with clinical repercussion, however the preoperative identification of this anomaly is important in case of ascending aorta surgery and may cause difficulties in catheterization during angiography. Most frequently, it occurs in the right coronary artery, sometimes in association with a bicuspid aortic valve^(8)^ (Figure 7).

### Coronary ostium outside of the aortic coronary sinus

#### Anomalous origination of the coronary artery from the pulmonary arterial trunk

The origination of the coronary artery from the pulmonary artery constitutes one of the most severe anomalies, with clinical manifestations generally occurring at the first weeks of life, and death of 90% of affected children at their first year of life if left untreated^(2,4,8)^. It is among the differential diagnoses for marked cardiomegaly in the neonatal period^(8)^.

The most common presentation is the left coronary trunk originating from the pulmonary artery and the right coronary artery originating from the aorta (Bland-White-Garland syndrome)^(2)^. Such an anomaly leads to myocardial ischemia due to the coronary steal phenomenon, where the flow is redirected from the high-pressure system of the right coronary artery to the low-pressure pulmonary system by means of right coronary-left coronary collaterals^(4,8)^. In the literature, there are reports of late presentation of such syndrome in adults, probably caused by development of collaterals from the right coronary artery^(4)^. 

### Coronary ostium at improper coronary sinus

#### Right coronary originating from the left coronary sinus

**Figure 8:**
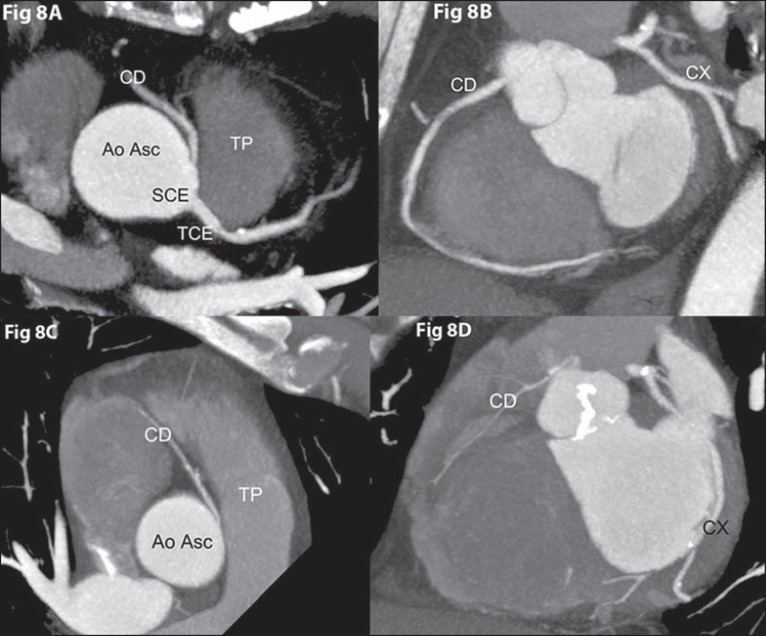
*Anomalous origination of right coronary artery from the left coronary sinus, with interarterial course*. **Fig 8A:** Axial coronary computed tomography angiography with MIP showing anomalous origination of right coronary artery (CD) from the left coronary sinus (SCE), with interarterial course between the pulmonary artery trunk (TP) and the ascending aorta (Ao Asc) (malignant course). **Fig 8B:** Same patient from **Fig 8A**, coronal MIP image of right coronary artery (CD) showing a large diameter artery with important anatomic and functional meaning. **Fig 8C:** Axial coronary computed tomography angiography with MIP showing another patient with anomalous origination of right coronary artery (CD) from the left coronary sinus and interarterial course between the pulmonary artery trunk (TP), anteriorly, and ascending aorta (Ao ASC), posteriorly (malignant course). **Fig 8D:** Same patient from **Fig 8C**, coronal MIP image of right coronary artery (CD) showing a hypoplastic artery, without anatomic and functional meaning. CX, circumflex artery.

Right coronary artery originating from the left coronary sinus or as a branch of a single coronary artery is found in 0.03% to 0.17% of the individuals submitted to angiography^(2)^. The most common proximal pathway of the right coronary in such cases is interarterial, and can be associated with sudden cardiac death in up to 30% of patients^(2)^ (Figure 8).

#### Left coronary trunk originating from the right coronary sinus

**Figure 9:**
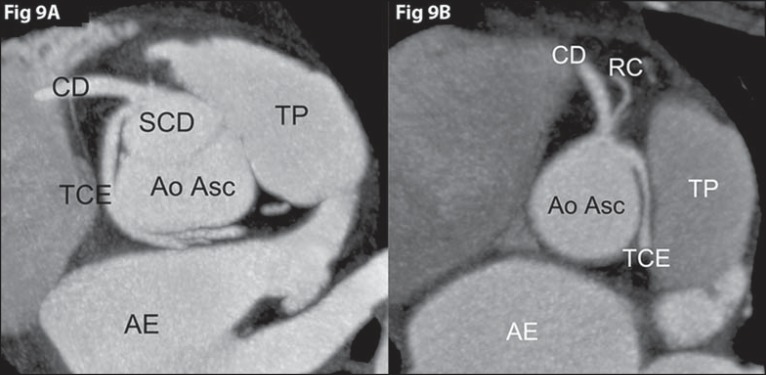
*Anomalous origination of left coronary trunk from the right coronary sinus*. **Fig 9A:** Benign course. Axial coronary computed tomography angiography with MIP showing anomalous origination of left coronary trunk (TCE) from the right coronary sinus (SCD), with retroaortic course between the ascending aorta (Ao Asc), anteriorly, and the left atrium (AE), posteriorly. **Fig 9B:** Malignant course. Axial coronary computed tomography angiography with MIP showing anomalous origination of the left coronary trunk (TCE) in the right coronary sinus (SCD), with interarterial course between the pulmonary artery trunk (TP), anteriorly, and the ascending aorta (Ao Asc), posteriorly. CD, right coronary artery; RC, conal branch; AE, left atrium.

Left coronary trunk originating from the right coronary sinus or as a branch of a single coronary artery occurs in 0.09% to 0.11% of the individuals submitted to angiography^(2)^. Proximal interarterial course occurs in 75% of such patients^(2)^ (Figure 9). 

#### Anterior descending or circumflex arteries originating from the right coronary sinus

**Figure 10:**
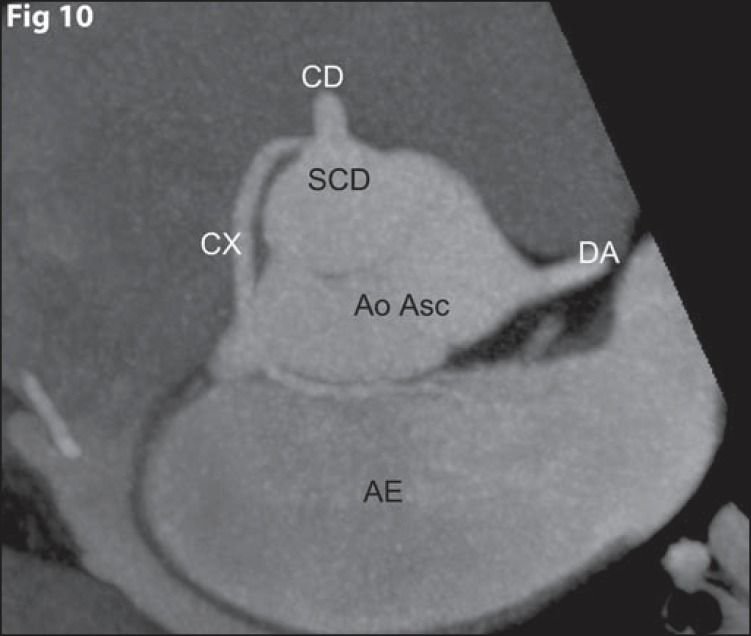
*Anomalous origination of the circumflex artery from the right coronary sinus, with benign course*. Axial coronary computed tomography angiography with MIP showing anomalous origination of the circumflex artery (CX) from the right coronary sinus (SCD), with retroaortic course between the ascending aorta (Ao Asc), anteriorly, and the left atrium (AE), posteriorly. The right coronary artery (CD) and the anterior descending artery (DA) have normal origination from the right and left coronary sinuses, respectively.

The circumflex artery is the one that most commonly presents anomalous origin, occurring in 0.32% to 0.67% of the population. Retroaortic pathway is its most common course, and there is no association with sudden death^(2)^ (Figure 10).

The anterior descending artery with anomalous origin rarely occurs in individuals with a normal cardiac anatomy. It is generally associated with Fallot’s tetralogy, complex transposition and double right ventricular output tract^(2)^. 

### Single coronary artery

**Figure 11:**
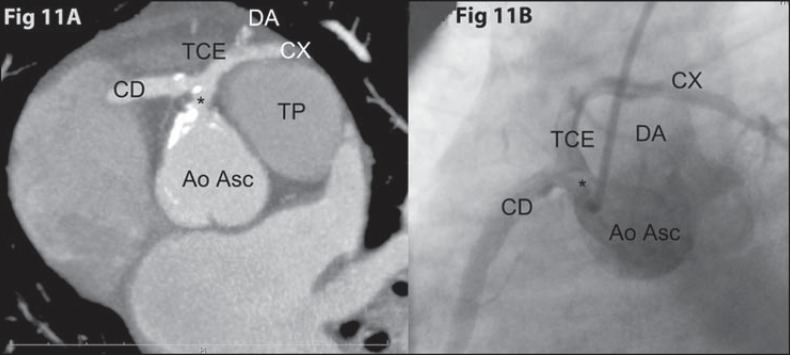
*Single coronary trunk*. **Fig 11A:** Oblique axial coronary computed tomography angiography with MIP showing single, short coronary trunk (asterisk) with calcified atheromatous plaque, originating from the right coronary sinus and giving origin to the right coronary artery (CD) and the left coronary trunk (TCE), which bifurcates into the anterior descending artery (DA) and the circumflex artery (CX), both travelling anteriorly to the pulmonary arterial trunk (TP). **Fig 11B:** Invasive coronary angiography (catheterization) identifying the same anatomical division demonstrated at coronary computed tomography angiography.

A single coronary artery originates from a single aortic root ostium. This is an extremely rare anomaly (0.0024% to 0.044% of the population) and may present with the pattern of a main trunk bifurcating into right coronary and left coronary trunk, one coronary artery originating as a branch from another with normal origin, or not following the habitual distribution of the coronary anatomy^(2)^ (Figure 11).

Such patients present with high risk for sudden death as the main trunk courses interarterialy. In addition, a proximal obstruction in the main trunk might be devastating, due to the unfeasibility of collateral circulation development.

### Anomalies of coronary artery termination

#### Congenital fistula

**Figure 12:**
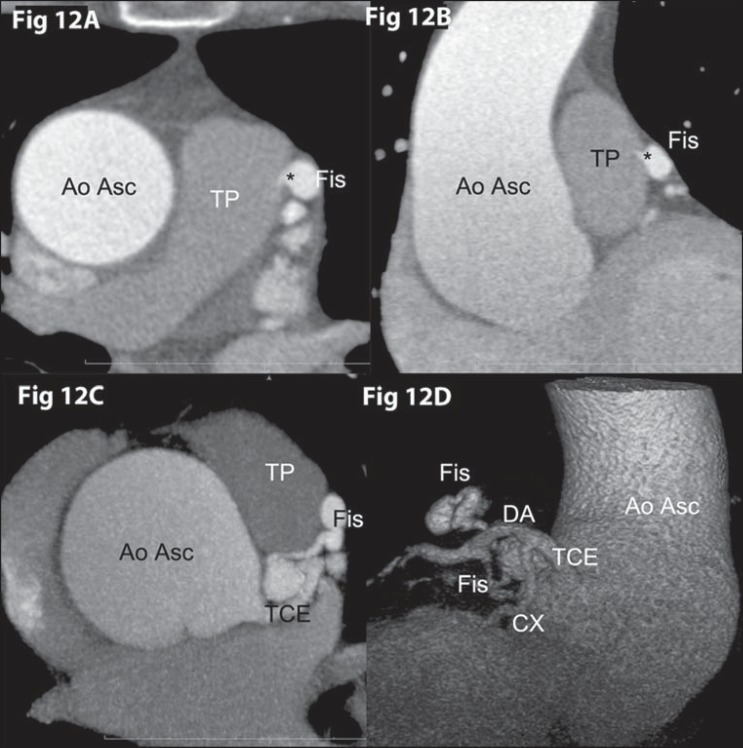
*Coronary fistula*. Coronary computed tomography angiography – axial (**Fig 11A**), oblique coronal (**Fig 11B**), axial MIP (**Fig 11C**) and volumetric rendering demonstrating a tortuous coronary fistula (* Fis), communicating the anterior descending artery (DA) and the pulmonary artery trunk (TP).

The normal coronary artery evetually branchs into a capillary bed in the myocardium. In cases where the coronary artery ends in a cardiac chamber or in a low-pressure vascular structure such as a pulmonary vessel, the steal phenomenon may occur, leading to inappropriate myocardial perfusion^(8)^ (Figure 12).

Termination in a chamber or low-pressure vessel may cause increase in caliber and tortuosity of the artery. In 60% of cases, it ends in the right chamber^(8)^.

### Symptoms of coronary anomalies

Most patients are asymptomatic, and the most frequent symptoms and signs include atypical chest pain, dyspnea, exercise-related syncope or pre-syncope, arrhythmia and left ventricular dysfunction^(1,6)^.

Patients above the age of 30 diagnosed with coronary anomalies in adulthood present a lower risk for sudden death, a fact that is, many times, taken into consideration in the therapeutic decision making process^(10)^.

As compared with normal arterial segments, coronary arteries with anomalous course are not more susceptible to obstructive atherosclerotic diseases^(5)^.

The risk for sudden death of athletes presenting with anomalous coronary artery origination is 79 times higher than in non-athlete individuals^(10)^.

### Diagnostic methods

Imaging methods are essential for the diagnosis of congenital coronary anomalies, since it is practically impossible to make a diagnosis by means of anamnesis, physical examination and electrocardiography, or even functional tests.

Transthoracic echocardiography presents limitations for such a characterization, especially as performed on adults and in the absence of a purpose-orientes study^(1,11)^. Brothers et al.^(12)^ suggest transthoracic echocardiography as a screening method for children and young adults who are first degree relatives of individuals who were victims of coronary anomalies-related sudden death.

Transesophageal echocardiography may be useful in the characterization of coronary arteries origination and proximal course^(9)^, but few reports are found in the literature in addition to fact that this is a semi-invasive method, not capable of demonstrating the entire pathway of such vessels^(13,14)^.

Angiography has already been considered to be the goldstandard method in such cases^(8,9)^, but it can hardly identify proximal course may be difficult, and is less accurate as compared with coronary computed tomography angiography (55% accuracy demonstrated in a study developed by Schmitt et al.)^(11,15,16)^.

Currently, computed tomography angiography or magnetic resonance imaging are considered as being the goldstandard to demonstrate the coronary anatomy^(6,11,17)^.

According to the American College of Cardiology Foundation appropriateness criteria for the utilization of cardiac computed tomography angiography^(18)^, such a method was considered “appropriate” (score 9/9) for investigation of patients with suspected coronary anomalies and has become a reference method^(8)^.

Computed tomography angiography detects not only the anomalous origination of such vessels, but also their course and relationship with other mediastinal structures, allowing for multiplanar and volumetric reformations, which play an essential role in the prognosis and evaluation for therapeutic approach^(1,4)^.

It is important to highlight that for the characterization of coronary anomalies, a specific protocol for computed tomography angiography with electrocardiographic synchronization is required, since in the absence of synchronization with heartbeats, pulse artifacts may generate images simulating an anomalous origination of the right coronary from the left coronary sinus with interarterial course^(16)^. 

The disadvantage of computed tomography as compared with magnetic resonance imaging is the utilization of ionizing radiation. New techniques have allowed for the reduction of radiation doses to even lower levels than those utilized in digital coronary angiography, while maintaining its excellent spatial resolution^(4)^.

Magnetic resonance imaging is also a good noninvasive method capable of demonstrating the coronary arteries origination and course, but its spatial resolution is significantly lower than that obtained by the new multidetector computed tomography apparatuses, in addition to its longer acquisition time^(19)^. The 3.0 T magnetic resonance imaging apparatuses have a signal-noise ratio approximately two times higher as compared with 1.5 T apparatuses, allowing for techniques with increased spatial resolution and shorter acquisition time^(20,21)^.

Functional diagnostic methods, such as myocardial scintigraphy or stress cardiac magnetic resonance imaging and intravascular ultrasonography, may be utilized to evaluate possible ischemia and/or associated myocardial fibrosis, assisting the therapeutic decision making^(1,6)^.

### Treatment

There are three treatment forms: 1) active surveillance/ drug treatment; 2) angioplasty with endoprosthesis implantation; 3) surgical treatment^(1)^.

Surgical treatment is generally the approach of choice for coronary anomalies of origination and course. However, the impact of such an approach on the survival of adult patients is still uncertain^(10)^.

A high number of authors indicate surgical treatment for anomalies of left coronary artery origination^(1,7,10-12)^. In cases of right coronary origination anomalies, the treatment is more controversial and is usually less agressive, depending on the clinical findings; and there are studies reporting a favorable evolution of some patients without surgical treatment^(11)^.

According to the American College of Cardiology and American Heart Association (ACC/AHA) recommendations published in 2008, surgical revascularization is indicated (class I) for the following conditions: 1) anomalous origination of left coronary trunk with interarterial course; 2) anomalous origination of right coronary artery with interarterial course in association with evidence of myocardial ischemia; 3) evidence of myocardial ischemia in the territory of the anomalous coronary artery without any other noticeable causal factor^(6)^.

Also, according to ACC/AHA recommendations, surgical revascularization could be beneficial (class IIa) in: 1) cases of significant stenosis demonstrated by intravascular ultrasonography; 2) vascular hypoplasia; 3) coronary artery compression or signs of coronary stenosis, even in the absence of proven association with ischemia^(6)^.

Surgical revascularization with vascular bypass/grafting is a widely utilized technique, sometimes with ligation of the native vessel due to the possibility of competitive flow development in this vessel, with consequential bypass/graft occlusion in some cases^(22)^. Other described techniques include: 1) unroofing, or fenestration of the intramural coronary segment, considered to be a simple and safe technique with reproducible results^(23)^; 2) ectopic artery reimplantation in the correct coronary sinus (technically difficult)^(1,7,10)^. Such techniques may yield better long-term outcomes, being utilized in children and in some adults^(22)^. Pulmonary artery translocation is also described as a means to avoid interarterial compression^(23)^.

The utilization of endoprosthesis has been described mainly for treatment of right coronary origination anomalies with proximal intramural segment stenosis^(1)^.

Treatment with beta-blockers is controversial and probably as effective as avoiding strenuous physical activities^(1)^.

## CONCLUSION 

Coronary computed tomography angiography has become the reference method in the evaluation of coronary anomalies. It is essential for the radiologists to recognize and characterize such anomalies and and their clinical significance.
